# Genetic Markers for Stevens-Johnson Syndrome/Toxic Epidermal Necrolysis in the Asian Indian Population: Implications on Prevention

**DOI:** 10.3389/fgene.2020.607532

**Published:** 2021-01-12

**Authors:** Swapna S. Shanbhag, Madhuri A. Koduri, Chitra Kannabiran, Pragnya R. Donthineni, Vivek Singh, Sayan Basu

**Affiliations:** ^1^The Cornea Institute, L.V. Prasad Eye Institute, Hyderabad, India; ^2^Brien Holden Eye Research Centre (BHERC), L.V. Prasad Eye Institute, Hyderabad, India; ^3^Manipal Academy of Higher Education, Manipal, India; ^4^Kallam Anji Reddy Molecular Genetics Laboratory, L.V. Prasad Eye Institute, Hyderabad, India; ^5^Center for Ocular Regeneration (CORE), L.V. Prasad Eye Institute, Hyderabad, India

**Keywords:** human leucocyte antigen, genetic markers, India, carbamazepine, anti-epileptics, toxic epidermal necrolysis, Stevens-Johnson Syndrome, severe cutaneous adverse drug reaction

## Abstract

This review attempts to collate all the studies performed in India or comprising a population originating from India and to find out if there is an association between the HLA (human leucocyte antigen) type of individual and development of Stevens-Johnson syndrome/toxic epidermal necrolysis (SJS/TEN) subsequent to medication use. The authors performed a PubMed search of all articles published in English from 2009 to 2019 for articles that studied HLA type in patients who developed SJS/TEN after intake of a specific drug in the Asian Indian population or in individuals of Asian Indian origin. The selection criteria were satisfied by a total of 11 studies that reported HLA associations with specific drugs, which induced SJS/TEN, mainly anti-epileptic drugs, and cold medicine/non-steroidal anti-inflammatory drugs. These studies involved a small number of patients, and hence, there is limited evidence to conclude if these associations can be extrapolated to a larger population of the same ethnicity. Similar multi-center studies need to be conducted with a larger sample size to confirm these associations. This would have implications in policy making and for understanding the potential of using genetic markers as a screening tool before prescribing a drug to a patient, which might make them susceptible to developing a potentially life-threatening disease such as SJS/TEN. This is possibly the only mode of primary prevention for this potentially fatal severe cutaneous adverse drug reaction.

## Introduction

Stevens-Johnson syndrome/toxic epidermal necrolysis (SJS/TEN) are diseases, which belong to a spectrum of immunological conditions affecting the skin and the mucosa. SJS/TEN are life-threatening conditions affecting multiple organ-systems, generally warrant intensive care unit or burn unit admission (Kohanim et al., 2016b; Shanbhag et al., 2020). Beginning as a skin rash and involvement of oral and ocular mucosa, it then evolves into necrolysis of the skin with involvement of mucosa of different organ systems (Kohanim et al., 2016a). Currently, supportive care guidelines are available for the treatment of SJS/TEN in the acute phase (Creamer et al., 2016; Seminario-Vidal et al., 2020). Despite aggressive treatment in the acute phase, the morbidity and mortality associated with SJS/TEN is still high (Hsu et al., 2016). On survival of the acute episode of SJS/TEN, a multitude of chronic complications affecting different organs still occur (Yang et al., 2016), the most debilitating of which are chronic ocular complications leading to corneal blindness (Saeed and Chodosh, 2016; Lee et al., 2017). Hence, primary prevention is the best form of prevention for SJS/TEN.

SJS/TEN is categorized as a severe cutaneous adverse reaction (SCAR) and multiple drugs have been implicated in the pathogenesis (Nguyen et al., 2019). Prevention is possible if patients who are susceptible to this SCAR on being prescribed a certain medication are identified. If a strong association is identified between ingestion of a drug and an HLA (human leukocyte antigen) type, then genetic screening of all patients before prescribing this drug to prevent the onset of SJS/TEN can be instituted. However, preemptive genotype screening before prescribing such medications is not yet practiced in the Asian Indian population due to the disease being uncommon and sparse evidence of such associations. This review was undertaken with the sole intention of understanding the existing evidence linking HLA associations with drug-induced SJS/TEN in the Asian Indian population.

## Data Sources and Selection Criteria

**Figure 1:**
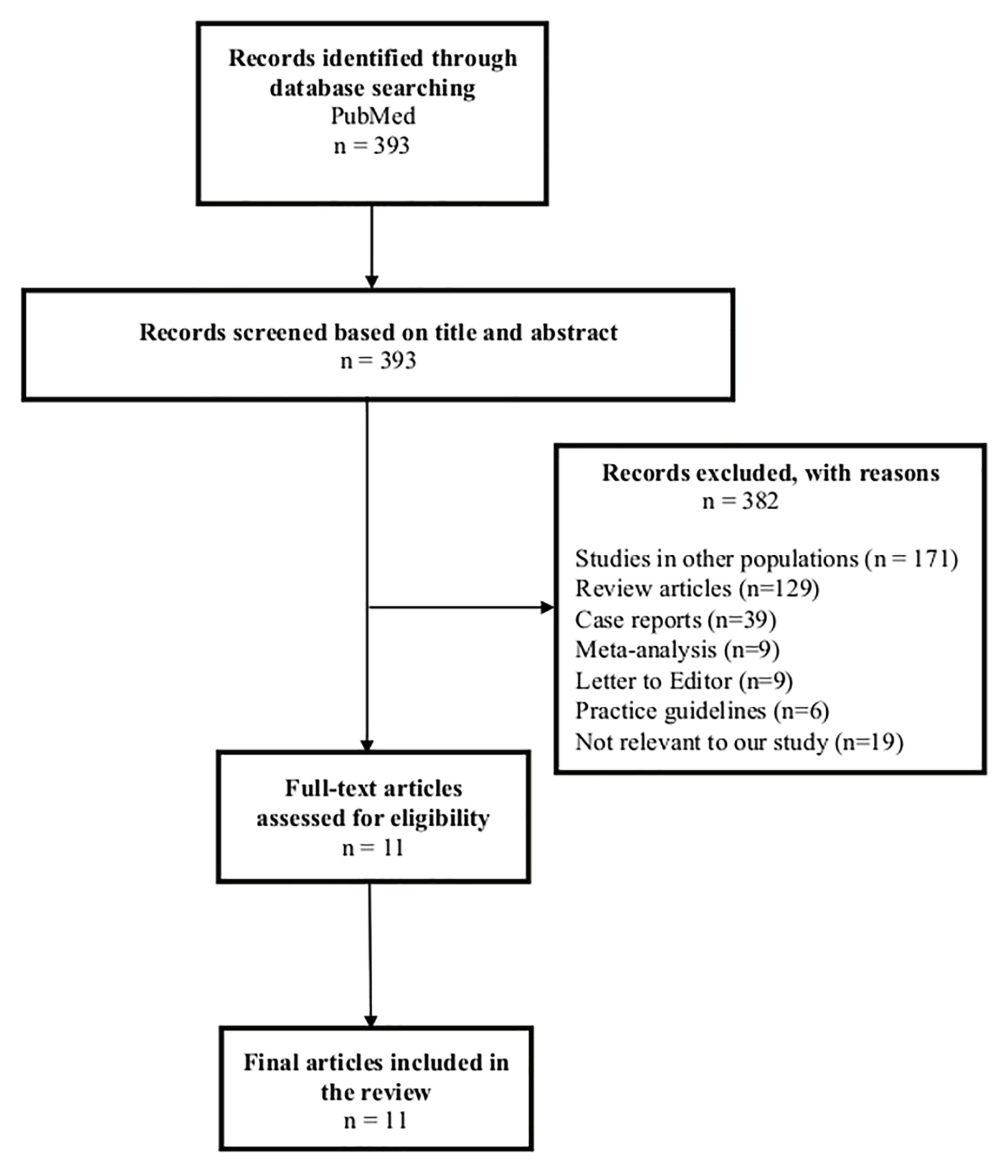
Flowchart of literature search for studies on genetic markers for Stevens-Johnson syndrome/toxic epidermal necrolysis in the Asian Indian population.

A search was conducted in February 2020 on PubMed for articles between January 1, 2009 and December 31, 2019. The keywords used were “Stevens-Johnson syndrome,” “toxic epidermal necrolysis,” “human leucocyte antigen,” “HLA,” “association,” “India,” and “Indian.” Articles in the English language were included. Letters, conference abstracts, case reports, review articles, editorials, and animal studies were excluded. A total of 393 articles were identified, out of which 382 articles did not fulfill inclusion criteria and were excluded after screening the titles and abstracts (Figure 1). A total of 11 studies met our criteria for inclusion and were further analyzed (Mehta et al., 2009; Chang et al., 2011; Aggarwal et al., 2014; Khor et al., 2014, 2017; Ueta et al., 2014; Ramanujam et al., 2016; Kannabiran et al., 2017; Srivastava et al., 2017; Devi, 2018; Ihtisham et al., 2019). Out of these 11 studies, three studies included a multi-ethnic population, including HLA associations in the Indian population in their countries (Chang et al., 2011; Khor et al., 2014, 2017).

### Associations Between Specific Drugs and HLA in the Pathogenesis of SJS/TEN

Most cases of SJS/TEN worldwide are associated with prior drug exposure. Certain drugs are implicated in the causation more than others such as antibacterial sulfonamides, carbamazepine (CBZ), allopurinol, lamotrigine, phenobarbital, phenytoin, nevirapine, and oxicam-type non-steroidal anti-inflammatory drugs (NSAIDs; Roujeau et al., 1995). SJS/TEN usually occurs the first time the drug is ingested, without prior sensitization, and usually within the first 2 months of therapy (Mockenhaupt et al., 2008).

The basis behind the pathogenesis of SJS/TEN is believed to be immunological. Genetic factors influencing drug metabolism and the immune response, including HLA genotype, might increase the risk of drug hypersensitivity, thus causing SJS/TEN (Roujeau et al., 1986; Svensson et al., 2001). The specific HLA allele presents a drug/metabolite to the T-cell receptors on cytotoxic T-lymphocytes resulting in cell activation, clonal expansion, and extensive keratinocyte death in SJS/TEN (Chung et al., 2008). This immunological hypothesis was given further credence by a study in the European population by Roujeau et al. (1987), where they noted that HLA alleles could be the primary genetic factor for determining an individual’s susceptibility to SJS/TEN. Further evidence for this finding was provided by Chung et al. (2004) in the Han-Chinese population, where presence of HLA-B*15:02 was strongly associated with CBZ-related SJS/TEN, and HLA-B*58:01 with allopurinol-related SJS/TEN (Hung et al., 2005b). However, HLA-B*15:02 was not significantly associated with CBZ-related SJS/TEN in the European population (Lonjou et al., 2006), which is explained by the low allele frequency of HLA-B*1502 of 1–2% in studies performed on European populations (Geer et al., 1998). This proves that HLA-B*15:02 is not a universal marker for CBZ-related SJS/TEN. Hence, it is important to study well-defined ethnic populations, identify the causative drug accurately, and test for specific HLA associations.

### Successful Implementation of Preemptive Genotyping for Prevention of SJS/TEN

The strongest association between HLA type and a drug causing SJS/TEN has been found between HLA-B*15:02 and CBZ in the Han-Chinese, Thai, and Malaysian populations (Chung et al., 2004; Tassaneeyakul et al., 2010; Chang et al., 2011; Tangamornsuksan et al., 2013), and HLA-B*58:01 and allopurinol in the Han-Chinese population (Hung et al., 2005b; Somkrua et al., 2011). The United States Food and Drug Administration in 2007 issued an alert regarding package labeling and recommended genotyping in all East Asian patients prior to prescribing CBZ (Ferrell and McLeod, 2008). Certain Asian countries, such as Taiwan, Hong Kong, Singapore, and Thailand, have since then started HLA-B*15:02 screening programs before the prescription of CBZ. This has been incorporated in the electronic prescribing system to interface with laboratory records to ensure that CBZ is not started in patients untested or positively tested for HLA-B*15:02. If patients screen positive for this allele, they are provided alternative medications. Certain countries like Thailand, Taiwan, and Singapore have included the cost of this screening in their national health insurance schemes (Dong et al., 2012; Tiamkao et al., 2013). In Taiwan, this measure clearly translated into a decrease in the incidence of CBZ-related SJS/TEN (Chen et al., 2011). Another study from Thailand also showed a significant decrease in the number of cases of CBZ-related SJS/TEN if a universal HLA-B*15:02 screening policy is instituted (Rattanavipapong et al., 2013). Regulatory recommendations for HLA-B*15:02 genotyping combined with government subsidy for the test also contributed to a reduction in CBZ-related SJS/TEN in Singapore by >90%, with additional reductions in number of phenytoin-related SJS/TEN cases (Sung et al., 2020).

### Cost-Effectiveness of Preemptive Genotyping for the Prevention of SJS/TEN

Calculating the cost-effectiveness of an intervention, such as preemptive genotyping, to reduce the incidence of SJS/TEN depends on several factors. These include the incidence and severity of the SCAR, the sensitivity and specificity of the marker, and the availability of inexpensive alternative medications with better safety profiles for individuals who screen positive for the marker (Chung et al., 2010). In the Han-Chinese population, the HLA-B*15:02 marker for CBZ-related SJS/TEN has been found to be 100% sensitive and 97% specific (Hung et al., 2005a). The average allele frequency of HLA-B*15:02 in the Han-Chinese population is 6% (1.9–12.4%; Gonzalez-Galarza et al., 2011). This is still relatively higher than other populations. Hence, screening for HLA-B*15:02 allele before starting treatment with CBZ in Asian countries is justified in view of high frequency of the allele, the seriousness of the consequences of SJS/TEN, high sensitivity and specificity of the marker as well as availability of alternative anti-epileptic drugs (AEDs; Chung et al., 2010). Whether screening prior to prescribing CBZ is financially viable depends on the extra cost of the test in a given population, whether the cost of the test is partially or fully covered by insurance, whether it outweighs the costs of SJS/TEN treatment, expense of alternative safer drugs, and subsequent loss of quality of life and due to sequelae of the ailment (Locharernkul et al., 2011). The second option is avoiding the use of CBZ altogether and prescribing alternative medications. However, CBZ use is still rampant in most South-East Asian countries since it is cheaper, effective, and physicians are experienced with its use (Chung et al., 2010). Alternative medications are more expensive preventing them from being cost-effective for the health-care system due to their long-term use (Locharernkul et al., 2011). Hence, countries in South-East Asia have consistently found that HLA-B*15:02 genotyping screening in the Han-Chinese population is less expensive than the cost of SJS/TEN treatment (both in the acute phase, in the chronic phase, including loss of quality-adjusted life years) or the cost of providing alternate drugs (Dong et al., 2012; Rattanavipapong et al., 2013; Tiamkao et al., 2013).

### Morbidity and Mortality of SJS/TEN in the Asian Indian Population

SJS/TEN is considered to be a rare condition with an estimated annual incidence (cases/million population/year) ranging from 0.6 to 12 cases per million population in different countries (Naldi et al., 1990; Schöpf et al., 1991; White et al., 2015; Hsu et al., 2016). Although the incidence of SJS/TEN in India is not known, it is possible that the incidence could be higher. Sushma et al. (2005) noted that 19.5% of hospitalized patients with SCAR over a 9-year period were diagnosed with SJS/TEN. A systematic review conducted on SJS/TEN in India reported an overall mortality of 12.94% in SJS/TEN cases (Patel et al., 2013), with the most common culprit drugs being antimicrobials (sulfonamides being the most common – 37%), followed by AEDs (CBZ and phenytoin being the most common – 36%), followed by NSAIDs (16%; Patel et al., 2013; Singh et al., 2015).

SJS/TEN contributes to life-long complications in the chronic phase, affecting multiple organ systems, with published reports from India discussing ophthalmic sequelae of SJS/TEN including bilateral corneal blindness (Kompella et al., 2002; Basu et al., 2018; Vazirani et al., 2018), respiratory and gastrointestinal system complications such as bronchiolitis obliterans (Basker et al., 1997; Dogra et al., 2014), esophageal strictures, drug-induced liver injury (Agrawal et al., 2003; Misra et al., 2004; Devarbhavi et al., 2016). Owing to the morbidity and mortality secondary to SJS/TEN in the Asian Indian population, measures in reducing the incidence of SJS/TEN could be beneficial in reducing the overall disease burden. Since HLA associations are not universal and are ethnicity specific, there is definitely a need to study if strong HLA genotype-drug associations in the Asian Indian population exist, thus making them more susceptible to developing SJS/TEN. Hence, a review of the existing literature on studies from India of HLA genotype-drug association on patients from the Asian Indian population who developed SJS/TEN to a specific drug was undertaken. Due to the paucity of such studies, studies performed on patients of Indian origin in countries other than India were also included.

## Results of HLA Genotyping in the Asian Indian Population with SJS/TEN

Descriptive information for each study is shown in Table 1. Out of the 11 studies, eight studies were from India, while three were from Malaysia. Among these eight studies, three included populations predominantly from North India (Aggarwal et al., 2014; Ramanujam et al., 2016; Ihtisham et al., 2019), and one study each included populations predominantly from South-India (Devi, 2018) and North-west India (Mehta et al., 2009). The studies conducted in Malaysia included a small cohort of Indian origin patients, predominantly from South India (Chang et al., 2011; Khor et al., 2014, 2017). The most commonly studied HLA genotype-drug association was HLA-B*15:02 and AEDs, specifically CBZ. Two studies focused on cold-medicine (CM) related SJS/TEN and studied HLA-A*02:06, HLA-A*33:03, and HLA-B*44:03. The number of patients tested in each study was small, ranging from 2 to 9 patients for the AED-related SJS/TEN, and 20–80 patients for CM-related SJS/TEN. All studies enrolled controls except one. Six studies enrolled controls that were drug-tolerant and had not developed SJS/TEN to AED’s, while four studies enrolled normal controls with no drug exposure. All studies performed polymerase chain reaction with sequence-specific primers for HLA antigens.

**Table 1:** Descriptive information of the studies on genetic markers for Stevens-Johnson syndrome/toxic epidermal necrolysis (SJS/TEN) in the Asian Indian population.

S.no	Author	Country	Allele studied	Number of Indian patients with SJS/TEN with allele	Number of controls with allele	Drug	Reported OR, 95% CI, *p* value
1	Mehta et al., 2009	Gujarat, Northwest India	HLA-B*15:02	6/8	0/10 normal controls	CBZ	71.40, 3.0–1,698, 0.0014
2	Chang et al., 2011	Malaysia (multi-ethnic population with Indians, predominantly South Indian)	HLA-B*15:02	2/2	47/300 normal controls from Malaysian population	CBZ	NA
3	Aggarwal et al., 2014	Chandigarh, North India	HLA-B*15:02	2/9 (CBZ) 0/8 (Phenytoin)	50 tolerant controls (0/37 on CBZ, 0/13 on phenytoin)	CBZ, PHT	NA, NA, 0.035
4	Khor et al., 2014	Malaysia (multi-ethnic population with Indians, predominantly South Indian)	HLA-B*15:02	2/5	52 Indian CBZ-tolerant controls	CBZ	16.7, 1.7–163, 0.0349
5	Ueta et al., 2014	India	HLA-A*02:06 HLA-B*44:03	1/20 12/20	3/55 normal controls 6/55 normal controls	Cold medicine^¶^ (multi-ingredient cold medications and NSAIDs)	0.91, 0.09–9.06, 0.939; 10.88, 4.04–29.3, 1.87.E-07
6	Ramanujam et al., 2016	New Delhi, North India	HLA-B*15:02	1/2	25 LEV-tolerant controls	LEV	NA
7	Khor et al., 2017	Malaysia (multi-ethnic population with Indians, predominantly South Indian)	HLA-B*15:02 HLA-A*31:01	2/6 (HLA-B*15:02) 3/6 (HLA-A*31:01)	2/57 (HLA-B*15:02) 5/57 (HLA-A*31:01) All 57 Indian CBZ-tolerant controls	CBZ	13.8, 1.51–124.99, 0.04; 10.4, 1.64–65.79, 0.023
8	Srivastava et al., 2017	India	HLA-B*15:02	1/3	NA	Lamotrigine	NA
9	Kannabiran et al., 2017	India	HLA-A*33:03 HLA-B*44:03 HLA-C*07:01	37/80 (HLA-A*33:03) 50/80 (HLA-B*44:03) 47/80 (HLA-C*07:01)	10/50 (HLA-A*33:03) 6/50 (HLA-B*44:03) 9/50 (HLA-C*07:01) All 50 normal controls	Cold medicine^ǂ^	2.8, 1.4–5.5, 2.9.E-02; 10.1, 4.4–23.1, 1.3.E-09; 6.2, 3.0–12.7, 6.9.E-07
10	Devi, 2018	Kerala, South India	HLA-B*15:02	1/4 (CBZ), 0/8 (phenytoin)	11 tolerant controls (0/3 on CBZ, 0/18 on phenytoin)	CBZ, PHT	NA
11	Ihtisham et al., 2019	New Delhi, North India	HLA-B*57:01 HLA-DRB1*07:01	2/5 in CBZ-induced SJS/TEN cases (HLA-B*57:01) 3/5 in CBZ-induced SJS/TEN cases (HLA-DRB1*07:01)	4/70 CBZ-tolerant positive for HLA-B*57:01; 12/70 CBZ-tolerant controls positive for HLA-DRB1*07:01	CBZ	11.00, 1.41–85.81, 0.05; 7.25, 1.09–48.18, 0.01

*HLA, human leucocyte antigen; CBZ, carbamazepine; PHT, phenytoin; LEV, levetiracetam; NSAIDs, non-steroidal anti-inflammatory drugs; and NA, not available*.

¶ *The exact drug etiology for SJS/TEN was not known in all patients; specific phenotype of patients with severe ocular complications in the chronic phase of SJS/TEN were studied*.

ǂ *The exact drug etiology for SJS/TEN were known in 28.8% (23/80) patients; specific phenotype of patients with severe ocular complications in the chronic phase of SJS/TEN were studied*.

### Results of Studies of Associations Between HLA Alleles and Anti-Epileptic Drugs in the Indian Population With SJS/TEN

The studies performed in India in Table 1 show that HLA-B*15:02 remains a significant risk predictor of CBZ-related SJS/TEN. The average allele frequency of HLA-B*15:02 in the Indian population among different communities evaluated primarily from the North Indian population is 2.5% (0–6%; Rajalingam et al., 2002; Rani et al., 2007). Since the carrier frequency in the Indian population is lower than the Han-Chinese population, there is a need to test for other susceptibility genes for CBZ-related SJS/TEN in the Indian population. In a study by Khor et al. (2017), HLA-A*31:01 was found to be associated significantly with CBZ-related SJS/TEN in Indians. In another study by Ihtisham et al. (2019), HLA-B*57:01 was found to be associated significantly with CBZ-related SJS/TEN in Indians. The allele frequency of HLA-A*31:01 and HLA-B*57:01 in the Indian population among different communities is 3.52% (primarily from South India) and 2–8% (from both North and South India; Gonzalez-Galarza et al., 2011), respectively. Although the frequencies of these alleles are similar to the allele frequency of HLA-B*15:02 in the Indian population, the utility of testing these alleles have not been otherwise studied widely for CBZ-related SJS/TEN. Collection of data from multiple centers will be useful as samples collected from individual centers may prove to be too small to attain a statistical significance. Also, the carrier frequency for HLA-B*15:02 may not be homogenously distributed in the Indian population, and multiple studies across the length and the breadth of the country are required to establish this, in the normal population.

CBZ is a commonly used AED in India because of affordability and easy availability and most studies on HLA type-drug association in SJS/TEN have focused on this drug. However, few studies have tested for susceptibility genes for SJS/TEN caused by other aromatic AED’s (phenytoin and lamotrigine) in the Indian population (Aggarwal et al., 2014; Srivastava et al., 2017; Devi, 2018). However, the prescribing patterns for these drugs in India are not yet known, and hence, it is unclear if preemptive genotyping for these will prove to be useful and cost-effective. Also, it is essential to enroll controls that are tolerant to the drug in these studies to enable the study of cost-effectiveness of using such a test for screening.

Guidelines are available, provided by the Clinical Pharmacogenetics Implementation Consortium (CPIC) for appropriate usage of drugs like CBZ, oxcarbazepine, and phenytoin, which are some of the main culprit drugs for SJS/TEN (Leckband et al., 2013; Caudle et al., 2014; Phillips et al., 2018; Karnes et al., 2020). These guidelines provide therapeutic recommendations on how these drugs need to be utilized when genotyping results are available. Since these guidelines greatly assist clinicians in applying genetic information to patient care, thus optimizing the therapeutic usage of these drugs, physicians who routinely prescribe these drugs should be aware of these guidelines.

### Results of Studies of Associations Between HLA Alleles and Cold Medicines (NSAIDs) in the Indian Population

Two studies studied associations between CM-related SJS/TEN and HLA-A*02:06, HLA-A*33:03, HLA-B*44:03, and HLA-C*07:01 in the Asian Indian population (Ueta et al., 2014; Kannabiran et al., 2017). Patients with the specific phenotype of severe ocular complications (SOC) in the chronic phase were selected for these studies, although exact drug etiology for SJS/TEN in all cases was not known in both studies. The details of these are mentioned in Table 1. In patients with SOC in the chronic phase with CM-related SJS/TEN, an association has been noted between HLA-A*02:06 in Japanese and Koreans, HLA-B*44:03 in Indian, Brazilian Caucasians, Thai, and Japanese populations, and HLA-C*07:01 in the Indian and Thai population (Ueta, 2015; Jongkhajornpong et al., 2018). Also, a significant genome-wide association between CM-related SJS/TEN and *IKZF1* SNPs (single nucleotide polymorphisms) were noted in the Japanese, Korean, Indian, and Thai populations with severe mucosal involvement (SMI), suggesting that IKZF1 might be a potential marker for susceptibility to CM-related SJS/TEN with SMI (Ueta et al., 2015; Chantaren et al., 2019). The genotypes of the associated SNP in the *IKZF1* gene reflected a quantitative difference in the ratio of transcripts of the gene produced by alternative splicing (Ueta et al., 2015).

However, preemptive genotyping before prescribing cold medications may not be feasible as these are commonly prescribed drugs and are available over-the-counter (Tangamornsuksan et al., 2020). Ascribing the cause of SJS/TEN to cold medications is problematic due to protopathic bias, where NSAIDs may be given to patients for the prodromal symptoms, which occur when SJS/TEN has already set in but is yet to evolve into a full-blown disease (Horwitz and Feinstein, 1980; Roujeau et al., 2018). Hence, the ALDEN (assessment of drug causality for epidermal necrolysis) algorithm comes into play here, where strict guidelines are followed to find out if a certain drug caused SJS/TEN so as not to create a situation, where drugs that might not have caused SJS/TEN are labeled so and have to be avoided (Sassolas et al., 2010). Strict definitions are required for labeling the day of disease-onset (Kelly et al., 1995). With AED’s, there is credible evidence that they are known to cause SJS/TEN (Roujeau et al., 1995; Mockenhaupt et al., 2008). However, the same amount of evidence for cold-medications, such as salicylates, ibuprofen, and acetaminophen, causing SJS/TEN does not exist (Mockenhaupt et al., 2008; Lebrun-Vignes et al., 2018). Also, most studies that have found a genetic association between CM-related SJS/TEN and HLA type have been in the population of SJS/TEN patients in the chronic phase (Ueta, 2015), which predisposes these studies to a recall bias. Using the ALDEN algorithm in the chronic phase may not be accurate unless rigorous documentation is available. Thus, it may be difficult to conclude that NSAIDs were the primary reason for SJS/TEN, especially in patients on multiple medications. Roujeau et al. (2018) suggested that it is possible that idiopathic SJS/TEN or SJS/TEN caused due to infections such as *Mycoplasma pneumoniae* could be labeled CM-related SJS/TEN if the ALDEN algorithm is not rigorously followed.

In a systematic review from India, NSAIDs were found to be responsible in 16% cases, cold-medications among these constituted 55% of total NSAIDs causing SJS/TEN (Patel et al., 2013). However, no drug causality algorithms were used to deduce this information. Also, self-medication with NSAIDs is common due to the availability of these over-the-counter (OTC; Doomra and Goyal, 2020). In a recent study from India, NSAIDs were found to be the second most common causative factor for SCAR (Thakkar et al., 2017). However, the information regarding causative drugs, especially NSAIDs in this study was not available in one-fourth cases due to use of OTC medications and absence of documentation. Hence, preemptive genotyping for these drugs may not be practical.

## Drawbacks of Preemptive Genotyping Based on Hla Associations

One possible drawback of preemptive genotyping is highlighted by Chen et al. (2016) in a study, where they evaluated the cost-effectiveness of pharmacogenetic screening. They noted that HLA-B*15:02 screening policy in Hong Kong has not been cost-effective due to a shift in prescription from CBZ to alternate AED’s, an increase in SJS/TEN caused by phenytoin intake post a policy to implement screening, poor adherence to the policy (Chen et al., 2014), an unwillingness of clinicians to wait for the screening results before prescribing alternative AED’s causing an increase in expenditure by screening but this not being translated to immediate benefits of screening. The unwillingness to wait for the screening test’s result was due to a long-turnaround time for the result and the need for an additional consultation to be scheduled. Clinicians preferred prescribing phenytoin as no genetic screening was required, this led to an increase in SJS/TEN caused by phenytoin, such that the overall burden of AED-induced SJS/TEN was unchanged (Chen et al., 2014). Chen et al. (2016) suggested that the cost-effectiveness of implementing this screening test may be improved by enhancing policy adherence by clinicians, making clinicians aware of SJS/TEN caused by other AED’s, and by less expensive rapid point-of-care genotyping. Full genotyping may be expensive and specific allele typing may be more practical and cost-effective. Testing for specific HLA alleles, including HLA-B*15:02 should be made easily accessible and economical. At present, the expected cost of single HLA genotyping in India is approximately 80–100 USD with a turnaround time of 2–3 weeks, making this test expensive combined with a long-waiting time to decide if the drug can be prescribed.

Using an alternative, safer AED without the need to performing genotyping is another form of reducing costs (Locharernkul et al., 2011). However, this requires more research on safety profiles of different AED’s. Although prevention of SJS/TEN will benefit a patient with high-risk of developing it, it is not clear whether the additional cost of screening will be covered by insurers, employers or the national health care systems (Locharernkul et al., 2011).

Also, HLA genotype may not be the only predictive factor for the development of SJS/TEN. Other than HLA genotype, factors such as initial drug dosing and renal function tests could also impact the risk of drug-induced SJS/TEN (Stamp et al., 2012; Ramasamy et al., 2013; Chung et al., 2015). For example, for allopurinol-induced SCAR, HLA-B*58:01 allele is not absolutely necessary or sufficient to explain the disease. The positive predictive value is estimated to be 2.7%, implying that other risk factors may be involved in the pathogenesis (Lonjou et al., 2008; Tassaneeyakul et al., 2009; Chung et al., 2015). Hence, in conjunction with HLA genotyping, further investigations are required to explain the role of HLA in predicting the development of SJS/TEN.

## Directions For Future Research

Further research is required in finding the true incidence rates of SJS/TEN in India, preferably *via* a registry-based approach. The most common causative drugs that cause SJS/TEN in India need to be ascertained nation-wide. Prescribing patterns of these drugs need to be studied to be able to quantify the risk of SJS/TEN with the use of such medications. Physicians should be made aware of the pharmacogenomics of SJS/TEN and availability of preemptive genotyping. Physicians should also be made aware of CPIC guidelines for appropriate therapeutic usage of drugs that commonly cause SJS/TEN, when genotyping results are available.

Genotyping for specific HLA associations could be made more accessible, less expensive, with rapid results.

More studies need to be conducted in the normal population in various communities across the country in order to ascertain the prevalence of certain HLA alleles implicated in the development of SJS/TEN. Next, studies need to be conducted, preferably *via* a multi-centric approach in patients with SJS/TEN after exposure to a certain drug, to find if an HLA association exists. However, such studies first need to establish drug causality stringently. Once these factors are taken into consideration, certain policy recommendations can be instituted. Prevention of SJS/TEN may be possible by the integration of an effective pharmacovigilance system into routine health care.

## Conclusions

Although SJS/TEN is considered as a rare disease, the burden of the disease is great with high degrees of morbidity and mortality with severe long-term sequelae in survivors affecting multiple organ systems. These create a substantial economic burden for the patient as well as the caregivers. Further research on primary prevention of this dreaded disease is necessary.

## Author Contributions

SS: concept and design of study. SS and MK: literature search and interpretation of data. SS, MK, CK, PD, VS, and SB: drafting the article or revising it critically for important intellectual content and final approval of the version to be published. All authors contributed to the article and approved the submitted version.

### Conflict of Interest

The authors declare that the research was conducted in the absence of any commercial or financial relationships that could be construed as a potential conflict of interest.

## References

[ref1] Aggarwal R. Sharma M. Modi M. Garg V. K. Salaria M. (2014). HLA-B*1502 is associated with carbamazepine induced Stevens-Johnson syndrome in north Indian population. Hum. Immunol. 75, 1120–1122. 10.1016/j.humimm.2014.09.022, PMID: 25305458

[ref2] Agrawal A. Bramble M. G. Shehade S. Dean J. (2003). Oesophageal stricturing secondary to adult Stevens-Johnson syndrome: similarities in presentation and management to corrosive injury. Endoscopy 35, 454–457. 10.1055/s-2003-38765, PMID: 12701021

[ref3] Basker M. Cherian T. Raghupathy P. (1997). Chronic lung disease following Stevens-Johnson syndrome. Indian Pediatr. 34, 831–835. PMID: 9492425

[ref4] Basu S. Shanbhag S. S. Gokani A. Kedar R. Bahuguna C. Sangwan V. S. (2018). Chronic ocular sequelae of Stevens-Johnson syndrome in children: long-term impact of appropriate therapy on natural history of disease. Am J. Ophthalmol. 189, 17–28. 10.1016/j.ajo.2018.01.028, PMID: 29421293

[ref5] Caudle K. E. Rettie A. E. Whirl-Carrillo M. Smith L. H. Mintzer S. Lee M. T.. (2014). Clinical pharmacogenetics implementation consortium guidelines for CYP2C9 and HLA-B genotypes and phenytoin dosing. Clin. Pharmacol. Ther. 96, 542–548. 10.1038/clpt.2014.159, PMID: 25099164 PMC4206662

[ref6] Chang C. C. Too C. L. Murad S. Hussein S. H. (2011). Association of HLA-B*1502 allele with carbamazepine-induced toxic epidermal necrolysis and Stevens-Johnson syndrome in the multi-ethnic Malaysian population. Int. J. Dermatol. 50, 221–224. 10.1111/j.1365-4632.2010.04745.x, PMID: 21244392

[ref7] Chantaren P. Jongkhajornpong P. Ueta M. Puangsricharern V. Lekhanont K. Pisuchpen P.. (2019). Association of IKZF1 SNPs in cold medicine-related Stevens-Johnson syndrome in Thailand. Clin. Transl. Allergy 9:61. 10.1186/s13601-019-0300-9, PMID: 31768251 PMC6873726

[ref8] Chen P. Lin J. J. Lu C. S. Ong C. T. Hsieh P. F. Yang C. C.. (2011). Carbamazepine-induced toxic effects and HLA-B*1502 screening in Taiwan. N. Engl. J. Med. 364, 1126–1133. 10.1056/NEJMoa1009717, PMID: 21428768

[ref9] Chen Z. Liew D. Kwan P. (2014). Effects of a HLA-B*15:02 screening policy on antiepileptic drug use and severe skin reactions. Neurology 83, 2077–2084. 10.1212/WNL.0000000000001034, PMID: 25355835

[ref10] Chen Z. Liew D. Kwan P. (2016). Real-world cost-effectiveness of pharmacogenetic screening for epilepsy treatment. Neurology 86, 1086–1094. 10.1212/WNL.0000000000002484, PMID: 26888992

[ref11] Chung W. H. Chang W. C. Stocker S. L. Juo C. G. Graham G. G. Lee M. H.. (2015). Insights into the poor prognosis of allopurinol-induced severe cutaneous adverse reactions: the impact of renal insufficiency, high plasma levels of oxypurinol and granulysin. Ann. Rheum. Dis. 74, 2157–2164. 10.1136/annrheumdis-2014-205577, PMID: 25115449

[ref12] Chung W. H. Hung S. I. Chen Y. T. (2010). Genetic predisposition of life-threatening antiepileptic-induced skin reactions. Expert Opin. Drug Saf. 9, 15–21. 10.1517/14740330903427969, PMID: 20001755

[ref13] Chung W. H. Hung S. I. Hong H. S. Hsih M. S. Yang L. C. Ho H. C.. (2004). Medical genetics: a marker for Stevens-Johnson syndrome. Nature 428:486. 10.1038/428486a, PMID: 15057820

[ref14] Chung W. H. Hung S. I. Yang J. Y. Su S. C. Huang S. P. Wei C. Y.. (2008). Granulysin is a key mediator for disseminated keratinocyte death in Stevens-Johnson syndrome and toxic epidermal necrolysis. Nat. Med. 14, 1343–1350. 10.1038/nm.1884, PMID: 19029983

[ref15] Creamer D. Walsh S. A. Dziewulski P. Exton L. S. Lee H. Y. Dart J. K. G.. (2016). UK guidelines for the management of Stevens-Johnson syndrome/toxic epidermal necrolysis in adults 2016. J. Plast. Reconstr. Aesthet. Surg. 69, e119–e153. 10.1016/j.bjps.2016.01.034, PMID: 27287213

[ref16] Devarbhavi H. Raj S. Aradya V. H. Rangegowda V. T. Veeranna G. P. Singh R.. (2016). Drug-induced liver injury associated with Stevens-Johnson syndrome/toxic epidermal necrolysis: patient characteristics, causes, and outcome in 36 cases. Hepatology 63, 993–999. 10.1002/hep.28270, PMID: 26439084

[ref17] Devi K. (2018). The association of HLA B*15:02 allele and Stevens-Johnson syndrome/toxic epidermal necrolysis induced by aromatic anticonvulsant drugs in a south Indian population. Int. J. Dermatol. 57, 70–73. 10.1111/ijd.13812, PMID: 29076187

[ref18] Dogra S. Saini A. G. Suri D. Rawat A. Sodhi K. S. Singh S. (2014). Bronchiolitis obliterans associated with Stevens-Johnson syndrome and response to azathioprine. Indian J. Pediatr. 81, 732–733. 10.1007/s12098-013-1204-7, PMID: 24014187

[ref19] Dong D. Sung C. Finkelstein E. A. (2012). Cost-effectiveness of HLA-B*1502 genotyping in adult patients with newly diagnosed epilepsy in Singapore. Neurology 79, 1259–1267. 10.1212/WNL.0b013e31826aac73, PMID: 22955130

[ref20] Doomra R. Goyal A. (2020). NSAIDs and self-medication: a serious concern. J. Family Med. Prim. Care 9, 2183–2185. 10.4103/jfmpc.jfmpc_201_20, PMID: 32754470 PMC7380783

[ref21] Ferrell P. B. Jr. McLeod H. L. (2008). Carbamazepine, HLA-B*1502 and risk of Stevens-Johnson syndrome and toxic epidermal necrolysis: US FDA recommendations. Pharmacogenomics 9, 1543–1546. 10.2217/14622416.9.10.1543, PMID: 18855540 PMC2586963

[ref22] Geer L. Terasaki P. I. Gjertson D. W. (1998). “Histocompatibility, T. P. E. H. L. A. S. F. & and immunogenetics” in HLA frequency. eds. Gjertson D. W. Terasaki P. I. (Lenexa: American Society for Histocompatibility and Immunogenetics).

[ref23] Gonzalez-galarza F. F. Christmas S. Middleton D. Jones A. R. (2011). Allele frequency net: a database and online repository for immune gene frequencies in worldwide populations. Nucleic Acids Res. 39, D913–D919. 10.1093/nar/gkq1128, PMID: 21062830 PMC3013710

[ref24] Horwitz R. I. Feinstein A. R. (1980). The problem of “protopathic bias” in case-control studies. Am. J. Med. 68, 255–258. 10.1016/0002-9343(80)90363-0, PMID: 7355896

[ref25] Hsu D. Y. Brieva J. Silverberg N. B. Silverberg J. I. (2016). Morbidity and mortality of Stevens-Johnson syndrome and toxic epidermal necrolysis in United States adults. J. Invest. Dermatol. 136, 1387–1397. 10.1016/j.jid.2016.03.023, PMID: 27039263

[ref26] Hung S. I. Chung W. H. Chen Y. T. (2005a). HLA-B genotyping to detect carbamazepine-induced Stevens-Johnson syndrome: implications for personalizing medicine. Per. Med. 2, 225–237. 10.2217/17410541.2.3.225, PMID: 29793265

[ref27] Hung S. I. Chung W. H. Liou L. B. Chu C. C. Lin M. Huang H. P.. (2005b). HLA-B*5801 allele as a genetic marker for severe cutaneous adverse reactions caused by allopurinol. Proc. Natl. Acad. Sci. U. S. A. 102, 4134–4139. 10.1073/pnas.0409500102, PMID: 15743917 PMC554812

[ref28] Ihtisham K. Ramanujam B. Srivastava S. Mehra N. K. Kaur G. Khanna N.. (2019). Association of cutaneous adverse drug reactions due to antiepileptic drugs with HLA alleles in a north Indian population. Seizure 66, 99–103. 10.1016/j.seizure.2019.02.011, PMID: 30826555

[ref29] Jongkhajornpong P. Lekhanont K. Pisuchpen P. Chantaren P. Puangsricharern V. Prabhasawat P.. (2018). Association between HLA-B*44:03-HLA-C*07:01 haplotype and cold medicine-related Stevens-Johnson syndrome with severe ocular complications in Thailand. Br. J. Ophthalmol. 102, 1303–1307. 10.1136/bjophthalmol-2017-311823, PMID: 29706602

[ref30] Kannabiran C. Ueta M. Sangwan V. Rathi V. Basu S. Tokunaga K.. (2017). Association of human leukocyte antigen class 1 genes with Stevens Johnson syndrome with severe ocular complications in an Indian population. Sci. Rep. 7:15960. 10.1038/s41598-017-15965-7, PMID: 29162886 PMC5698496

[ref31] Karnes J. H. Rettie A. E. Somogyi A. A. Huddart R. Fohner A. E. Formea C. M.. (2020). Clinical Pharmacogenetics Implementation Consortium (CPIC) guideline for CYP2C9 and HLA-B genotypes and phenytoin dosing: 2020 update. Clin. Pharmacol. Ther. 10.1002/cpt.2008, PMID: [Epub ahead of print] 32779747 PMC7831382

[ref32] Kelly J. P. Auquier A. Rzany B. Naldi L. Bastuji-garin S. Correia O.. (1995). An international collaborative case-control study of severe cutaneous adverse reactions (SCAR). Design and methods. J. Clin. Epidemiol. 48, 1099–1108. 10.1016/0895-4356(95)00004-n, PMID: 7636511

[ref33] Khor A. H. Lim K. S. Tan C. T. Kwan Z. Tan W. C. Wu D. B.. (2017). HLA-A*31: 01 and HLA-B*15:02 association with Stevens-Johnson syndrome and toxic epidermal necrolysis to carbamazepine in a multiethnic Malaysian population. Pharmacogenet. Genomics 27, 275–278. 10.1097/FPC.0000000000000287, PMID: 28570299

[ref34] Khor A. H. Lim K. S. Tan C. T. Wong S. M. Ng C. C. (2014). HLA-B*15:02 association with carbamazepine-induced Stevens-Johnson syndrome and toxic epidermal necrolysis in an Indian population: a pooled-data analysis and meta-analysis. Epilepsia 55, e120–e124. 10.1111/epi.12802, PMID: 25266342

[ref35] Kohanim S. Palioura S. Saeed H. N. Akpek E. K. Amescu A. G. Basu S. (2016a). Acute and chronic ophthalmic involvement in Stevens-Johnson syndrome/toxic epidermal necrolysis—a comprehensive review and guide to therapy. II. Ophthalmic disease. Ocul. Surf. 14, 168–188. 10.1016/j.jtos.2016.02.001, PMID: 26882981

[ref36] Kohanim S. Palioura S. Saeed H. N. Akpek E. K. Amescua G. Basu S.. (2016b). Stevens-Johnson syndrome/toxic epidermal necrolysis—a comprehensive review and guide to therapy. I. Systemic disease. Ocul. Surf. 14, 2–19. 10.1016/j.jtos.2015.10.002, PMID: 26549248

[ref37] Kompella V. B. Sangwan V. S. Bansal A. K. Garg P. Aasuri M. K. Rao G. N. (2002). Ophthalmic complications and management of Stevens-Johnson syndrome at a Tertiary Eye Care Centre in South India. Indian J. Ophthalmol. 50, 283–286. PMID: 12532492

[ref39] Lebrun-Vignes B. Guy C. Jean-Pastor M. J. Gras-Champel V. Zenut M. (2018). Is acetaminophen associated with a risk of Stevens-Johnson syndrome and toxic epidermal necrolysis? Analysis of the French Pharmacovigilance database. Br. J. Clin. Pharmacol. 84, 331–338. 10.1111/bcp.13445, PMID: 28963996 PMC5777438

[ref40] Leckband S. G. Kelsoe J. R. Dunnenberger H. M. George A. L. Jr. Tran E. Berger R.. (2013). Clinical Pharmacogenetics Implementation Consortium guidelines for HLA-B genotype and carbamazepine dosing. Clin. Pharmacol. Ther. 94, 324–328. 10.1038/clpt.2013.103, PMID: 23695185 PMC3748365

[ref41] Lee H. Y. Walsh S. A. Creamer D. (2017). Long-term complications of Stevens-Johnson syndrome/toxic epidermal necrolysis (SJS/TEN): the spectrum of chronic problems in patients who survive an episode of SJS/TEN necessitates multidisciplinary follow-up. Br. J. Dermatol. 177, 924–935. 10.1111/bjd.15360, PMID: 28144971

[ref42] Locharernkul C. Shotelersuk V. Hirankarn N. (2011). Pharmacogenetic screening of carbamazepine-induced severe cutaneous allergic reactions. J. Clin. Neurosci. 18, 1289–1294. 10.1016/j.jocn.2010.12.054, PMID: 21802305

[ref43] Lonjou C. Borot N. Sekula P. Ledger N. Thomas L. Halevy S.. (2008). A European study of HLA-B in Stevens-Johnson syndrome and toxic epidermal necrolysis related to five high-risk drugs. Pharmacogenet. Genomics 18, 99–107. 10.1097/FPC.0b013e3282f3ef9c, PMID: 18192896

[ref44] Lonjou C. Thomas L. Borot N. Ledger N. De Toma C. Lelouet H.. (2006). A marker for Stevens-Johnson syndrome …: ethnicity matters. Pharmacogenomics J. 6, 265–268. 10.1038/sj.tpj.6500356, PMID: 16415921

[ref45] Mehta T. Y. Prajapati L. M. Mittal B. Joshi C. G. Sheth J. J. Patel D. B.. (2009). Association of HLA-B*1502 allele and carbamazepine-induced Stevens-Johnson syndrome among Indians. Indian J. Dermatol. Venereol. Leprol. 75, 579–582. 10.4103/0378-6323.57718, PMID: 19915237

[ref46] Misra S. P. Dwivedi M. Misra V. (2004). Esophageal stricture as a late sequel of Stevens-Johnson syndrome in adults: incidental detection because of foreign body impaction. Gastrointest. Endosc. 59, 437–440. 10.1016/s0016-5107(03)02710-x, PMID: 14997151

[ref47] Mockenhaupt M. Viboud C. Dunant A. Naldi L. Halevy S. Bouwes Bavinck J. N.. (2008). Stevens-Johnson syndrome and toxic epidermal necrolysis: assessment of medication risks with emphasis on recently marketed drugs. The EuroSCAR-study. J. Invest. Dermatol. 128, 35–44. 10.1038/sj.jid.5701033, PMID: 17805350

[ref48] Naldi L. Locati F. Marchesi L. Cainelli T. (1990). Incidence of toxic epidermal necrolysis in Italy. Arch. Dermatol. 126, 1103–1104. 10.1001/archderm.1990.01670320127028, PMID: 2383037

[ref49] Nguyen D. V. Vidal C. Chu H. C. Van Nunen S. (2019). Human leukocyte antigen-associated severe cutaneous adverse drug reactions: from bedside to bench and beyond. Asia Pac. Allergy 9:e20. 10.5415/apallergy.2019.9.e20, PMID: 31384575 PMC6676067

[ref50] Patel T. K. Barvaliya M. J. Sharma D. Tripathi C. (2013). A systematic review of the drug-induced Stevens-Johnson syndrome and toxic epidermal necrolysis in Indian population. Indian J. Dermatol. Venereol. Leprol. 79, 389–398. 10.4103/0378-6323.110749, PMID: 23619444

[ref51] Phillips E. J. Sukasem C. Whirl-carrillo M. Müller D. J. Dunnenberger H. M. Chantratita W.. (2018). Clinical Pharmacogenetics Implementation Consortium guideline for HLA genotype and use of carbamazepine and oxcarbazepine: 2017 update. Clin. Pharmacol. Ther. 103, 574–581. 10.1002/cpt.1004, PMID: 29392710 PMC5847474

[ref52] Rajalingam R. Krausa P. Shilling H. G. Stein J. B. Balamurugan A. McGinnis M. D.. (2002). Distinctive KIR and HLA diversity in a panel of north Indian Hindus. Immunogenetics 53, 1009–1019. 10.1007/s00251-001-0425-5, PMID: 11904677

[ref53] Ramanujam B. Ihtisham K. Kaur G. Srivastava S. Mehra N. K. Khanna N.. (2016). Spectrum of cutaneous adverse reactions to levetiracetam and human leukocyte antigen typing in north-Indian patients. J. Epilepsy Res. 6, 87–92. 10.14581/jer.16016, PMID: 28101480 PMC5206105

[ref54] Ramasamy S. N. Korb-wells C. S. Kannangara D. R. Smith M. W. Wang N. Roberts D. M.. (2013). Allopurinol hypersensitivity: a systematic review of all published cases, 1950–2012. Drug Saf. 36, 953–980. 10.1007/s40264-013-0084-0, PMID: 23873481

[ref55] Rani R. Marcos C. Lazaro A. M. Zhang Y. Stastny P. (2007). Molecular diversity of HLA-A, -B and -C alleles in a north Indian population as determined by PCR-SSOP. Int. J. Immunogenet. 34, 201–208. 10.1007/s40264-013-0084-0, PMID: 17504510

[ref56] Rattanavipapong W. Koopitakkajorn T. Praditsitthikorn N. Mahasirimongkol S. Teerawattananon Y. (2013). Economic evaluation of HLA-B*15:02 screening for carbamazepine-induced severe adverse drug reactions in Thailand. Epilepsia 54, 1628–1638. 10.1111/epi.12325, PMID: 23895569

[ref57] Roujeau J. C. Bracq C. Huyn N. T. Chaussalet E. Raffin C. Duédari N. (1986). HLA phenotypes and bullous cutaneous reactions to drugs. Tissue Antigens 28, 251–254. 10.1111/j.1399-0039.1986.tb00491.x, PMID: 3544335

[ref58] Roujeau J. C. Dunant A. Mockenhaupt M. (2018). Epidermal necrolysis, ocular complications, and “Cold Medicines”. J Allergy Clin Immunol Pract 6, 703–704. 10.1016/j.jaip.2017.10.033, PMID: 29525000

[ref59] Roujeau J. C. Huynh T. N. Bracq C. Guillaume J. C. Revuz J. Touraine R. (1987). Genetic susceptibility to toxic epidermal necrolysis. Arch. Dermatol. 123, 1171–1173. 3477129

[ref60] Roujeau J. C. Kelly J. P. Naldi L. Rzany B. Stern R. S. Anderson T.. (1995). Medication use and the risk of Stevens-Johnson syndrome or toxic epidermal necrolysis. N. Engl. J. Med. 333, 1600–1607. 10.1056/NEJM199512143332404, PMID: 7477195

[ref61] Saeed H. N. Chodosh J. (2016). Ocular manifestations of Stevens-Johnson syndrome and their management. Curr. Opin. Ophthalmol. 27, 522–529. 10.1097/ICU.0000000000000312, PMID: 27585215

[ref62] Sassolas B. Haddad C. Mockenhaupt M. Dunant A. Liss Y. Bork K.. (2010). ALDEN, an algorithm for assessment of drug causality in Stevens-Johnson syndrome and toxic epidermal necrolysis: comparison with case-control analysis. Clin. Pharmacol. Ther. 88, 60–68. 10.1038/clpt.2009.252, PMID: 20375998

[ref63] Schöpf E. Stühmer A. Rzany B. Victor N. Zentgraf R. Kapp J. F. (1991). Toxic epidermal necrolysis and Stevens-Johnson syndrome. An epidemiologic study from West Germany. Arch. Dermatol. 127, 839–842. 10.1001/archderm.1991.01680050083008, PMID: 2036029

[ref64] Seminario-Vidal L. Kroshinsky D. Malachowski S. J. Sun J. Markova A. Beachkofsky T. M.. (2020). Society of Dermatology Hospitalists supportive care guidelines for the management of Stevens-Johnson syndrome/toxic epidermal necrolysis in adults. J. Am. Acad. Dermatol. 82, 1553–1567. 10.1016/j.jaad.2020.02.066, PMID: 32151629

[ref65] Shanbhag S. S. Chodosh J. Fathy C. Goverman J. Mitchell C. Saeed H. N. (2020). Multidisciplinary care in Stevens-Johnson syndrome. Ther. Adv. Chronic Dis. 11, 2040622319894469. 10.1177/2040622319894469 32523661 PMC7236394

[ref66] Singh P. K. Kumar M. K. Kumar D. Kumar P. (2015). Morphological pattern of cutaneous adverse drug reactions due to antiepileptic drugs in eastern India. J. Clin. Diagn. Res. 9, WC01–WC03. 10.7860/JCDR/2015/11701.5419, PMID: 25738068 PMC4347159

[ref67] Somkrua R. Eickman E. E. Saokaew S. Lohitnavy M. Chaiyakunapruk N. (2011). Association of HLA-B*5801 allele and allopurinol-induced Stevens Johnson syndrome and toxic epidermal necrolysis: a systematic review and meta-analysis. BMC Med. Genet. 12:118. 10.1186/1471-2350-12-118, PMID: 21906289 PMC3189112

[ref68] Srivastava S. Ramanujam B. Ihtisham K. Tripathi M. (2017). Cutaneous adverse drug reactions to lamotrigine and human leukocyte antigen typing in north Indian patients: a case series. Ann. Indian Acad. Neurol. 20, 408–410. 10.4103/aian.AIAN_234_17, PMID: 29184346 PMC5682747

[ref69] Stamp L. K. Taylor W. J. Jones P. B. Dockerty J. L. Drake J. Frampton C.. (2012). Starting dose is a risk factor for allopurinol hypersensitivity syndrome: a proposed safe starting dose of allopurinol. Arthritis Rheum. 64, 2529–2536. 10.1002/art.34488, PMID: 22488501

[ref71] Sung C. Tan L. Limenta M. Ganesan G. Toh D. Chan C. l. (2020). Usage pattern of carbamazepine and associated severe cutaneous adverse reactions in Singapore following implementation of HLA-B*15:02 genotyping as standard-of-care. Front. Pharmacol. 11:527. 10.3389/fphar.2020.00527, PMID: 32457602 PMC7221117

[ref72] Sushma M. Noel M. V. Ritika M. C. James J. Guido S. (2005). Cutaneous adverse drug reactions: a 9-year study from a South Indian Hospital. Pharmacoepidemiol. Drug Saf. 14, 567–570. 10.1002/pds.1105, PMID: 15937869

[ref73] Svensson C. K. Cowen E. W. Gaspari A. A. (2001). Cutaneous drug reactions. Pharmacol. Rev. 53, 357–379. PMID: 11546834

[ref74] Tangamornsuksan W. Chaiyakunapruk N. Somkrua R. Lohitnavy M. Tassaneeyakul W. (2013). Relationship between the HLA-B*1502 allele and carbamazepine-induced Stevens-Johnson syndrome and toxic epidermal necrolysis: a systematic review and meta-analysis. JAMA Dermatol. 149, 1025–1032. 10.1001/jamadermatol.2013.4114, PMID: 23884208

[ref75] Tangamornsuksan W. Chanprasert S. Nadee P. Rungruang S. Meesilsat N. Ueta M.. (2020). HLA genotypes and cold medicine-induced Stevens-Johnson syndrome/toxic epidermal necrolysis with severe ocular complications: a systematic review and meta-analysis. Sci. Rep. 10:10589. 10.1038/s41598-020-67610-5, PMID: 32601360 PMC7324363

[ref76] Tassaneeyakul W. Jantararoungtong T. Chen P. Lin P. Y. Tiamkao S. Khunarkornsiri U. (2009). Strong association between HLA-B*5801 and allopurinol-induced Stevens-Johnson syndrome and toxic epidermal necrolysis in a Thai population. Pharmacogenet. Genomics 19, 704–709. 10.1097/FPC.0b013e328330a3b8, PMID: 19696695

[ref77] Tassaneeyakul W. Tiamkao S. Jantararoungtong T. Chen P. Lin S. Y. Chen W. H.. (2010). Association between HLA-B*1502 and carbamazepine-induced severe cutaneous adverse drug reactions in a Thai population. Epilepsia 51, 926–930. 10.1111/j.1528-1167.2010.02533.x, PMID: 20345939

[ref78] Thakkar S. Patel T. K. Vahora R. Bhabhor P. Patel R. (2017). Cutaneous adverse drug reactions in a Tertiary Care Teaching Hospital in India: an Intensive Monitoring Study. Indian J. Dermatol. 62, 618–625. 10.4103/ijd.IJD_703_16, PMID: 29263536 PMC5724310

[ref79] Tiamkao S. Jitpimolmard J. Sawanyawisuth K. Jitpimolmard S. (2013). Cost minimization of HLA-B*1502 screening before prescribing carbamazepine in Thailand. Int. J. Clin. Pharm. 35, 608–612. 10.1007/s11096-013-9777-9, PMID: 23649893

[ref80] Ueta M. (2015). Genetic predisposition to Stevens-Johnson syndrome with severe ocular surface complications. Cornea 34(Suppl 11), S158–S165. 10.1097/ICO.0000000000000605, PMID: 26448174

[ref81] Ueta M. Kannabiran C. Wakamatsu T. H. Kim M. K. Yoon K. C. Seo K. Y.. (2014). Trans-ethnic study confirmed independent associations of HLA-A*02:06 and HLA-B*44:03 with cold medicine-related Stevens-Johnson syndrome with severe ocular surface complications. Sci. Rep. 4:5981. 10.1038/srep05981, PMID: 25099678 PMC4124463

[ref82] Ueta M. Sawai H. Sotozono C. Hitomi Y. Kaniwa N. Kim M. K.. (2015). IKZF1, a new susceptibility gene for cold medicine-related Stevens-Johnson syndrome/toxic epidermal necrolysis with severe mucosal involvement. J. Allergy Clin. Immunol. 135:1538.e17–1545.e17. 10.1016/j.jaci.2014.12.1916, PMID: 25672763

[ref83] Vazirani J. Nair D. Shanbhag S. Wurity S. Ranjan A. Sangwan V. (2018). Limbal stem cell deficiency-demography and underlying causes. Am J. Ophthalmol. 188, 99–103. 10.1016/j.ajo.2018.01.020, PMID: 29378178

[ref84] White M. L. Chodosh J. Jang J. Dohlman C. (2015). Incidence of Stevens-Johnson syndrome and chemical burns to the eye. Cornea 34, 1527–1533. 10.1097/ICO.0000000000000646, PMID: 26488629

[ref85] Yang C. W. Cho Y. T. Chen K. L. Chen Y. C. Song H. L. Chu C. Y. (2016). Long-term sequelae of Stevens-Johnson syndrome/toxic epidermal necrolysis. Acta Derm. Venereol. 96, 525–529. 10.2340/00015555-2295, PMID: 26582440

